# Analysis of Immunological Characteristics and Genomic Alterations in HPV-Positive Oropharyngeal Squamous Cell Carcinoma Based on PD-L1 Expression

**DOI:** 10.3389/fimmu.2021.798424

**Published:** 2022-01-25

**Authors:** Sheng-ming Xu, Chao-ji Shi, Rong-hui Xia, Li-zhen Wang, Zhen Tian, Wei-min Ye, Liu Liu, Shu-li Liu, Chun-ye Zhang, Yu-hua Hu, Rong Zhou, Yong Han, Yu Wang, Zhi-yuan Zhang, Jiang Li

**Affiliations:** ^1^ Department of Oral and Maxillofacial-Head Neck Oncology, Ninth People’s Hospital, Shanghai Jiao Tong University School of Medicine, Shanghai, China; ^2^ National Clinical Research Center for Oral Diseases, National Center for Stomatology, Shanghai, China; ^3^ Shanghai Key Laboratory of Stomatology & Shanghai Research Institute of Stomatology, Shanghai, China; ^4^ Research Unit of Oral and Maxillofacial Regenerative Medicine, Chinese Academy of Medical Sciences, Shanghai, China; ^5^ Department of Oral Pathology, Shanghai Ninth People’s Hospital, College of Stomatology, Shanghai Jiao Tong University School of Medicine, Shanghai, China

**Keywords:** HPV, OPSCC, PD-L1, tumor immune microenvironment (TIM), genomic alterations

## Abstract

Programmed death-ligand 1 (PD-L1) expression has been approved as an immune checkpoint inhibitor (ICI) response predictive biomarker; however, the clinicopathological and molecular features of HPV-positive oropharyngeal squamous cell carcinoma [HPV(+)OPSCC] based on PD-L1 expression are not well studied. We aimed to characterize clinicopathological, tumor immune microenvironmental, and molecular features of HPV(+)OPSCC with different PD-L1 expression scored by combined positive score (CPS). A total of 112 cases were collected from 2008-2021 and received PD-L1 and CD8 immunohistochemistry (IHC) staining. 71 samples received DNA sequencing out of which 32 samples received RNA sequencing for immune-related gene alterations or expression analysis. The 32 samples were also subjected to analysis of CD20, CD4, CD8, CD68, Foxp3 and P16 by multiplex immunofluorescence (mIF) staining, and the immune markers were evaluated in the tumor body (TB), tumor margin (TM) and normal stroma (NS) regions separately. Our results showed that of 112 HPV(+)OPSCC tumors, high(CPS≥20), intermediate(1≤CPS<20), and low(CPS<1) PD-L1 expression was seen in 29.5%, 43.8% and 26.8% cases respectively. Non-smoking patients and patients with tumors occurring at the tonsils or having rich lymphocytes infiltration had significantly higher PD-L1 expression. Patients with CPS≥20 had significantly higher tumor mutation burden (TMB, p=0.0058), and PD-L1 expression correlated significantly with CD8^+^ T cells infiltration, which were ample in tumor regions than in NS in mIF. CD20^+^, CD4^+^, CD68^+^, Foxp3^+^CD4^+^ cells were demonstrated to infiltrate higher in TM while CD20^+^ and CD68^+^ cells were also enriched in NS and TB regions respectively. However, none of them showed correlations with PD-L1 expression. ARID1A, STK11 alterations were enriched in the low PD-L1 group significantly, while anti-viral immune associated APOBEC mutation signature and immune-related genes expression such as XCL1 and IL11 were positively associated with PD-L1 expression (p<0.05). This is a comprehensive investigation revealing immune and molecular features of HPV(+)OPSCC based on PD-L1 expression. Our study suggested that 73.2% of HPV(+)OPSCC patients may benefit from immunotherapy, and high PD-L1 expression reflects immune-active status of HPV(+)OPSCC accompanied by higher immune effect factors such as TMB, CD8^+^ cytotoxic T cells and immune-related genomic alterations. Our study offers valuable information for understanding the immune features of HPV(+)OPSCC.

## Introduction

As a biologically distinct entity among head and neck squamous cell cancers (HNSCCs), human papillomavirus (HPV)-positive oropharyngeal squamous cell carcinoma [HPV(+)OPSCC] has been assigned a specific staging algorithm in the 8th edition of the American Joint Committee on Cancer (AJCC) staging manual, indicating its unique prognosis and treatment strategy (1). Clinically, HPV(+)OPSCC is more prevalent in young males without a smoking history and shows sensitivity to chemoradiotherapy, resulting in significantly improved survival (2). Pathologically, HPV(+)OPSCC originates from the tonsil crypt epithelium rather than the surface epithelium, the former being surrounded by lymphoid follicles and highly infiltrated by immune cells, enhancing its antigen-capturing ability and immune surveillance (2). Additionally, the specific immune cell-infiltrated microenvironment and viral antigen confer a unique immunotherapy response to HPV(+)OPSCC, yielding insights into immunotherapy research.

The US Food and Drug Administration (FDA) has approved immune checkpoint inhibitors (ICIs), and specifically, programmed cell death protein 1 (PD-1) inhibitor pembrolizumab for recurrent and metastatic HNSCC based on the KEYNOTE-048 trial for which 882 participants were recruited (3, 4). In the trial, the expression of programmed cell death-ligand 1 (PD-L1) detected by monoclonal antibody 22C3 pharmDx (Agilent) showed the ability to predict the efficacy of pembrolizumab; therefore, the detection was approved by the FDA as a companion diagnosis for use of the PD-1 inhibitor (5). The trial illustrated distribution of PD-L1 combined positive score (CPS) in HNSCC and revealed that the patients with CPS ≥1 or CPS≥20 had better response to pembrolizumab and prolonged overall survival (5). Although 251 HPV(+)OPSCC were included in the trial, their CPS and the response to the therapy were not specifically indicated. Given the growing prevalence of HPV(+)OPSCC and the enormous potential of immunotherapy of the tumor, a comprehensive description of the tumor’s clinicopathological features based on PD-L1 expression is of significant importance.

Besides PD-L1, some other biomarkers have been reported as indicators of the efficacy of immunotherapy. Tumor mutation burden (TMB) and defects of mismatch repair (dMMR)/microsatellite instability (MSI) are meaningful genomic biomarkers for predicting ICI efficacy, and pembrolizumab has been approved by the FDA for treatment of unresectable/metastatic tumors with TMB≥10 mutation/Mb or dMMR/MSI-High regardless of tumor site or histology (6, 7). Pan-cancer research, however, found that TMB could only serve as an ICI response efficacy predictor when CD8^+^ T cells were positively associated with neoantigen load, which correlated positively with TMB, including melanoma and lung cancers, but not that of head and neck cancers (8), yet their HPV status were not clarified. Moreover, dMMR/MSI status in HPV(+)OPSCC still remained unclear. Therefore, illustrating TMB and dMMR/MSI status in HPV(+)OPSCC based on PD-L1 expression could increase our understating of immunotherapy resistance mechanisms or molecular features of this tumor.

Emerging evidence suggested that tumor infiltration lymphocyte (TIL) in tumor immune microenvironment (TIM) plays a vital role in tumor progression and immunotherapy resistance and shows chemotherapy response predictive ability in HNSCC (9). For example, CD8^+^ TILs combined with PD-L1 expression could predict better prognosis in HPV(+)OPSCC, and it was reported to be associated with treatment response to anti-PD-1/PD-L1 agents (10, 11), whereas little is known about the relationship between CD8^+^ TILs and PD-L1 in HPV(+)OPSCC. With the emergence of promising immunotherapy, it is important to study the interaction between TILs and PD-L1 expression. With tumor heterogeneity, different tumor regions exhibited distinct TILs, which reflected host immune response and predicted different clinical outcomes in colorectal cancer (12). Thus TILs should be investigated separately in different tumor regions. Additionally, genomic alterations including mutational signatures, driven gene mutations such as PTEN loss, MDM2 amplification, etc. as well as immune-related gene pathway expression have also been reported to be predictors of immunotherapeutic efficacy and to be associated with PD-L1 expression (13). Thus, the investigation of TILs in TIM, genomic alterations and immune-related gene expression profile of HPV(+)OPSCC based on PD-L1 CPS can help to better understand its molecular features with different PD-L1 levels and even immunotherapeutic effect or resistance mechanisms.

This study aimed to characterize clinicopathological, tumor immune microenvironmental and molecular features of HPV(+)OPSCC with different PD-L1 expression scored by CPS. The association between PD-L1 expression and other ICI efficacy biomarkers such as TMB, dMMR/MSI status was explored. Additionally, TIM including CD3^+^, CD4^+^, CD8^+^, CD20^+^, CD68^+^, Foxp3^+^CD4^+^ TILs infiltrations in different tumor regions and immune-related genomic alterations and gene expression of HPV(+)OPSCC stratified by PD-L1 CPS were also described using a cohort of 112 HPV(+)OPSCC cases in our institute from 2008–2021.

## Materials and Methods

### Patient Selection and Clinicopathological Information Collection

A total of 112 HPV(+)OPSCC samples from patients who received complete surgery were collected at the Shanghai 9^th^ People’s Hospital, Shanghai Jiaotong University from 2008 to 2021 with relevant clinical information, including age, gender, primary tumor sites, smoking and drinking status, lymph node status, TNM stage and pathological subtypes. We calculated each tumor’s TILs numbers and defined the top 50% as TIL-rich subtype, while other cases were defined as TIL-poor subtype. The patients’ HPV status were confirmed using the P16 immunohistochemistry (clone MX007, monoclonal, 1:100 dilution; Fuzhou Maixin Biotech. Co, Ltd., Fujian, China), HPV DNA PCR (PCR-RDB, Reverse Dot Blot; Yaneng BIO, Guangdong, China) and HPV RNA Scope HPV Kit (Advanced Cell Diagnostics, Inc., Hayword, CA). This study was considered exempt from the Independent Ethics Committee of the Shanghai 9th People’s Hospital, as it was carried out utilizing retrospective, deidentified clinical data. All cases were followed up until August 1st, 2021.

### Detecting and Scoring PD-L1 Expression

PD-L1 (22c3, DAKO, Santa Clara, CA) immunohistochemistry (IHC) assays were performed on full slides of formalin-fixed, paraffin-embedded (FFPE) HPV(+)OPSCC tissues using automated staining techniques with an auto-immuno0stainer following the manufacturer’s instructions. Positive and negative controls were used to monitor the quality of testing, and the results were independently reviewed by two pathologists from the Department of Oral Pathology, Shanghai 9th People’s Hospital (Jiang Li and Rong-hui Xia). CPS was calculated by dividing the number of PD-L1-positive cells (including tumor cells, lymphocytes, and macrophages) by the total number of viable tumor cells multiplied by 100, and any discrepancy was resolved by consensus after review. According to classification methodology A, PD-L1 subgroups were classified as low (CPS < 1, PD-L1-L), intermediate (1≤CPS < 20, PD-L1-M), and high (CPS ≥20, PD-L1-H). To assess patients’ stratification values of CPS=1 and CPS=20 as cut-off values, the whole group was classified as CPS<1 and CPS≥1 according to classification methodology B or as CPS<20 and CPS≥20 according to classification methodology C for analysis and comparison.

### CD8 and CD3 IHC and Calculation of CD8^+^ and CD3^+^ TILs Density

CD8 and CD3 IHC was performed for each case on a 4-mm tumor section cut from FFPE tissue blocks by using a monoclonal antibody against CD8 (clone 4B11,1 : 100 dilution, Novocastra, Newcastle, UK) and CD3 (clone LN10,1 : 100 dilution, Novocastra, Newcastle, UK) on an automated immunostainer. Case-viewer software (3DHISTECH.Ltd) was used to quantify total tumor area and percent of positive staining cells. The percentage of cells with weak, medium, and strong positive staining was determined using the color-deconvolution tool. CD8^+^ and CD3^+^ TILs density was calculated as the sum total of CD8^+^ and CD3^+^ cells (including those with weak, medium, and strong signals) divided by the area comprising the tumor and areas in direct contact with the tumor periphery (mm^2^).

### dMMR/MSI Testing

IHC for four MMR proteins (MLH1, PMS2, MSH2 and MSH6) was performed on the FFPE tissue of all cases. The antibody clones used were as follows: MLH-1 (clone ES05), MSH-2 (clone RED2), MSH-6 (clone UMAB258) and PMS-2 (clone EP51). All stains were performed on an auto-immuno-stainer according to manufacturer’s protocol. Normal crypt epithelium, lymphoid cells and stromal cells served as internal positive controls to exclude artefact and/or staining failure. Loss of any MMR proteins was defined as dMMR/MSI.

### Multiplex Immunofluorescence Staining and Visualization

The multiplex immunofluorescence (mIF) staining was performed step by step using Opal Polaris 7-color Manual IHC kit. Prepared slides were treated with microwave for 45 seconds at 100% power, and then covered with blocking buffer for 10 minutes. After draining off the blocking buffer, slides were incubated with a primary antibody working solution and then introduced with Opal Polymer HRP. The next steps included Opal signal amplification using the Opal working solution for 10 minutes followed by microwave treatment for 45 seconds. After repeating the steps above until all targets were detected except Opal Polaris 780, slides were blocked for 45 seconds, incubated with primary antibody according to manufacturer’s guidelines, and treated with the Opal TSA-TIG working solution for 10 minutes. After the second microwave treatment, the Opal Polaris 780 signal was generated using the Opal Polaris 780 working solution. The DAPI working solution was used for counterstaining and mounting. Antibodies against the following were used for staining: CD20 (Opal 570, yellow), CD68 (clone D4B9C, 1:200 dilution, Opal 520, green), CD4 (Opal 690, red), Foxp3 (clone D6O8R, 1:200 dilution, Opal 620, orange), CD8 (clone C8/144B, 1:200 dilution, Opal 480, blue), P16 (clone MX007, 1:100 dilution, Opal 780, purple) and DAPI for nuclei. Multiplex staining slides were imaged and scanned using the Vectra Polaris automated quantitative pathology imaging system. Fluorescence intensity information was extracted using the fluorescence protocol at 10 nm l from 420 nm to 780 nm by using the Akoya phenoptics inform software. After scanning the full image at low resolution, 15 regions of interest (ROI) from each slide were chosen by a pathologist (Jiang Li) using Phenochart 1.0.12 (Perkin Elmer), including 6 in the tumor body (TB), 6 at the tumor margin (TM), and 3 at the normal stroma (NS) area (defined in **Figures 3A, B**). The size of the ROIs was standardized at 2596560 pixels with a resolution of 0.497um/pixel for a total surface area of 0.642 mm^2^. TB, TM and NS area were defined according to **Figures 3A, B**. Immune cell populations were characterized and quantified using the cell segmentation and phenotype cell tool of the InForm image analysis software under pathologist’s supervision.

### DNA/RNA Sequencing and Analysis

Targeted DNA sequencing was performed for 36 FFPE samples, and then analyzed for TMB and genomic alterations. The amplified DNA was captured using the GenCap capture kit (MyGenostics Inc., Beijing, China). The enrichment libraries were sequenced on the Illumina HiSeq X 10 sequencer for paired-reading of 150 bp. The genomic alterations were detected by Varscan2 software. Whole-exome (WES) was performed on 35 tumors and matched normal samples. Each exome was captured using Agilent SureSelect V6 kit and sequenced on an Novaseq S4 PE150 to a depth of approximately 100 for germline and approximately 200 for tumor tissues. For DNA exome sequencing analysis, the trimmed and filtered readings were aligned to UCSC human reference genome (hg19) using BWA (v0.7.15) and further refined with the Picard (v2.18.7 tool. All somatic mutations were detected with each of the paired tumor–normal samples using Sention with the TNhaplotyper algorithm. All high-confident variants were annotated with Picard (v2.18.7) for consequence prediction and variant effect prediction. All somatic copy-number variant (CNV) determinations were carried out with a CNV kit (v0.9.3). Genes with copy number of at least three were defined as gain, and no more than one were defined as loss. The tumor mutation burden (TMB) was calculated by counting mutations in each sample meeting the following criteria: observed as PASS in TNhaplotyper, located within the targeted region, and absent in dbsnp and cosmic. Mutation counts were divided by target region size (35.08MB) giving mutation per megabase targeted. RNA sequencing (RNA-seq) was performed on 32 tumors. RNA libraries were prepared using the Truseq stranded mRNA and further sequenced on Novaseq S4 PE150. For RNA-sequencing (RNA-seq) data processing, Skewer (v0.2.2) was used to trim off adaptor containment sequences and low-quality bases at the ends. Trimmed and filtered reads were then aligned to the reference transcriptome (hg19) using STAR (2.4.2a). Gene-level quantification was conducted using RSEM(v1.2.29). Raw count data was normalized and further analyzed using the edgeR package. Gene expression values were log2 (1þx) transformed for downstream analysis. We selected a panel of immune-related markers (as shown in **Figure 7A**) and used ssGSEA to calculate immune cells infiltration score based on RNA-seq data for analysis and comparison.

### Statistical Analysis

The data were analyzed with SPSS statistical software program (SPSS, Statistics 26, IBM). The chi-square test was used to compare demographic and tumor-specific features in different CPS groups. Overall survival (OS) was defined as death from any cause, and OS was assessed by Kaplan–Meier analysis and evaluated using the log-rank test. Cox proportional hazards regression was used to examine the association between exposure factors and clinical outcomes. The Kruskal–Wallis test was used to compare and evaluate the distribution of different immune cells. All statistical tests were two-sided, and a P-value of 0.05 or less was considered to be statistically significant.

## Results

### Relationship Between PD-L1 Expression and Clinical Parameters in HPV(+)OPSCC and Overall Survival

A cohort of 112 HPV(+)OPSCC patients with relevant clinical information was selected for PD-L1 staining, which was subsequently scored using CPS and further analyzed. All cases were primary tumors, and all of the patients underwent surgery. **Table 1** shows that 26.8% (30 of 112) of patients had low PD-L1 expression (CPS<1), while 43.8% (49 of 112) had intermediate PD-L1 expression (1≤CPS<20), and 29.5% (33 of 112) had high PD-L1 expression (CPS≥20). The CPSs were significantly correlated with smoking status, primary tumor origin sites, and pathological subtypes but were not significantly correlated with age, sex, drinking status, TNM stage or lymph node metastasis status. Patients who had a history of smoking exhibited lower levels of PD-L1 expression when classified by methodologies A (p=0.032) and B (p=0.009). Tumors originating in the tonsils exhibited high levels of PD-L1 expression (p=0.02 by methodology A; p=0.012 by methodology B, and p=0.033 by methodology C), whereas the TIL-rich subtype tumors tended to express intermediate to high levels of PD-L1 (p<0.001), indicating the effects of unique HPV(+)OPSCC TIM on PD-L1 expression.

**Table 1:** Clinical characteristics of HPV (+)OPSCC classified by PD-L1 expression level.

CPS	<1 (30, 26.8%)	≥1,<20 (49, 43.8%)	≥20 (33, 29.5%)	Methodology A	Methodology B	Methodology C
Age	56.38	56.7	57.61	p = 0.82	p = 0.413	p=0.474
Gender						
Female	5 (20%)	15 (60%)	5 (20%)	p = 0.176	p = 0.385	p=0.239
Male	25 (28.7%)	34 (39.1%)	28 (32.2%)			
Sites						
Non-tonsil	28 (32.6%)	37 (43%)	21 (24.4%)	p = 0.02	p = 0.012	p=0.033
tonsil	2 (7.7%)	12 (46.2%)	12 (46.2%)			
Smoking Status						
No	14 (18.9%)	36 (48.6%)	24 (32.4%)	p = 0.032	p = 0.009	p=0.336
Yes	16 (42.1%)	13 (34.2%)	9 (23.7%)			
Drinking Status						
No	22 (24.4%)	41 (45.6%)	27 (30%)	p = 0.516	p = 0.258	p=0.801
Yes	8 (36.4%)	8 (36.4%)	6 (27.3%)			
TNM stage						
I	18 (22.5%)	39 (48.8%)	23 (28.7%)	p = 0.168	p = 0.096	p=0.758
II-III	12 (37.5%)	10 (31.3%)	10 (31.3%)			
LN Metastasis						
No	5 (31.3%)	6 (37.5%)	5 (31.3%)	p = 0.850	p = 0.663	p=0.866
Yes	25 (26%)	43 (44.8%)	28 (43.2%)			
Pathological Subtype						
TIL-poor	23 (41.1%)	24 (42.9%)	9 (16.1%)	p <0.001	p = 0.001	p=0.002
TIL-rich	7 (26.8%)	25 (43.8%)	24 (29.5%)			

**Figure 1:**
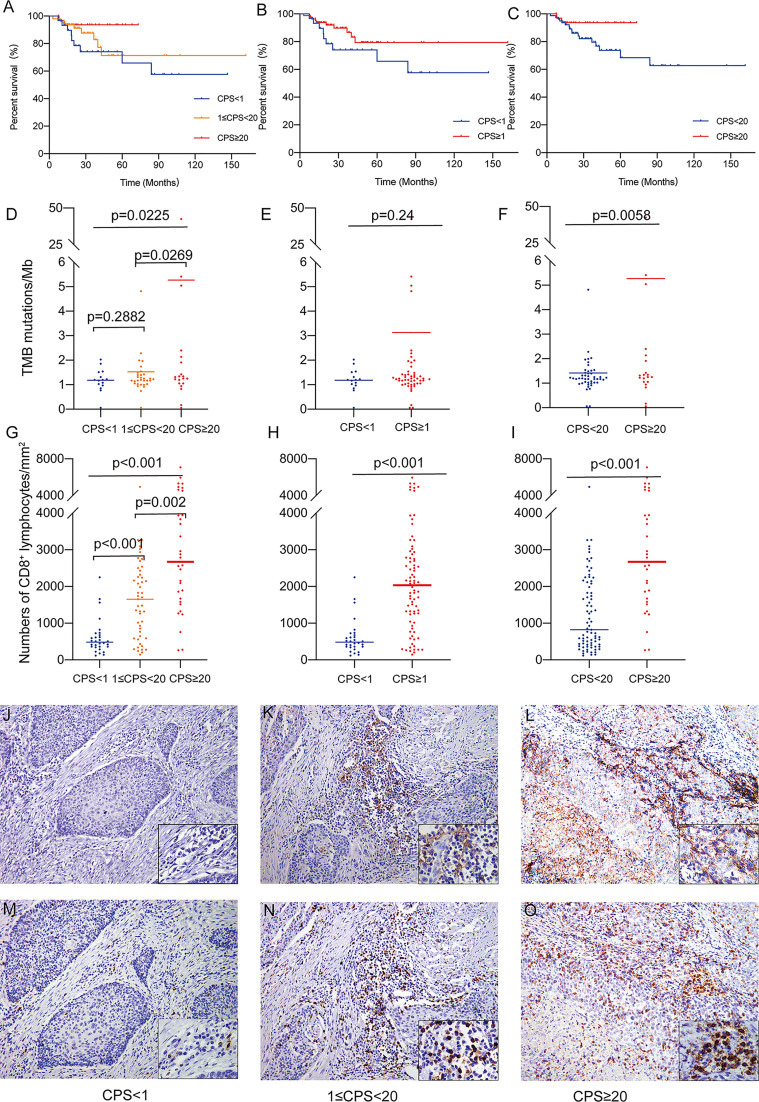
**(A)** Overall survival of patients classified by methodology A. **(B)** Overall survival of patients classified by methodology B. **(C)** Overall survival of patients classified by methodology C. **(D)** The association of TMB as a continuous variable and PD-L1 CPS classified by methodology A. **(E)** The association of TMB as a continuous variable and PD-L1 CPS classified by methodology B. **(F)** The association of TMB as a continuous variable and PD-L1 CPS classified by methodology C. **(G)** The association of CD8+ TILs density and PD-L1 CPS classified by methodology A. **(H)** The association of CD8+ TILs density and PD-L1 CPS classified by methodology B. **(I)** The association of CD8+ TILs density and PD-L1 CPS classified by methodology C. **(J)** PD-L1 staining (CPS<1, 200X). **(K)** PD-L1 staining (1<CPS≤20, 200X). **(L)** PD-L1 staining (CPS≥20, 200X). **(M)** CD8 staining (CPS<1, 200X). **(N)** CD8 staining (1<CPS≤20, 200X). **(O)** CD8 staining (CPS≥20, 200X).

All 112 patients had complete follow-up information available. In this study, the overall 5-year survival of HPV(+)OPSCC was 74.31%, whereas the 5-year overall survival of the CPS<1 group was 65.84%, and patients with a CPS ≥1 but<20 and CPS≥20 had 5-year survival rates of 71.30% and 93.65%, respectively. Defined by methodology A, B, or C, higher PD-L1 group showed a better prognosis trend although the difference was marginally significant as determined by the log-rank test (p=0.07 as classified by methodology A, p=0.14 as classified by methodology B, p=0.12 as classified by methodology C, **Figures 1A–C**).

### Association of the PD-L1 CPS With the TMB, CD8^+^ TILs Infiltration and dMMR/MSI in HPV(+)OPSCC

DNA sequencing and TMB analysis were performed on 71 samples from our cohort of 112 HPV(+)OPSCC patients. Overall, TMB increased with rising CPS levels, with the PD-L1-H group having significantly higher TMB than the PD-L1-L and PD-L1-M groups (5.268 vs. 1.186 and 1.527 mutations/Mb, p=0.0225, **Figure 1D**), but there was no significant difference between the PD-L1-L and PD-L1-M groups. Moreover, there was also no significant difference when classified according to methodology B (1.186 vs. 3.130 mutations/Mb, p=0.24, **Figure 1E**); however, the CPS≥20 group had significantly higher TMB than the CPS<20 groups as classified by methodology C (5.268 vs. 1.418 mutations/Mb, p=0.0058, **Figure 1F**), supporting the use of CPS≥20 as the cut-off value for a prospective patient selection strategy.

The CD8^+^ TILs infiltration in various groups was compared for all the patients. CD8^+^ TILs density ranged from 116 cells/mm^2^ to 7974 cells/mm^2^ and was positively correlated with CPS level (Spearman’s correlation coefficient=0.549, p<0.01). In the overall analysis, the density of CD8^+^ T lymphocyte infiltration was significantly elevated in the PD-L1-H group compared to the PD-L1-M and PD-L1-L groups (PD-L1-L, 606 cells/mm^2^; PD-L1-M, 1634 cells/mm^2^; PD-L1-H, 2973 cells/mm^2^; p< 0.001, **Figure 1G**). A significant difference in CD8^+^ TILs infiltration persisted regardless of whether the cohort was classified as CPS<1 and CPS≥1 (606 cells/mm^2^ vs. 2173 cells/mm^2^ p<0.001, **Figure 1H**) or as CPS<20 and CPS≥20 (1243 cells/mm^2^ vs. 2973 cells/mm^2^, p<0.001, **Figure 1I**). The typical images of PD-L1 and CD8 staining were illustrated in **Figures 1J–O**. TMB and CD8^+^ TILs density had a significant association (Pearson’s correlation coefficient=0.285, p=0.016, **Supplementary Figure S1**). In addition, the overall dMMR/MSI rate for all patients detected by MLH1, MSH2, MSH6, and PMS2 IHC was only 2.7% (3/112), and no association was observed between dMMR/MSI status and PD-L1 expression (data not shown).

### TIL Analysis Based on CPS Level by mIF Staining

**Figure 2:**
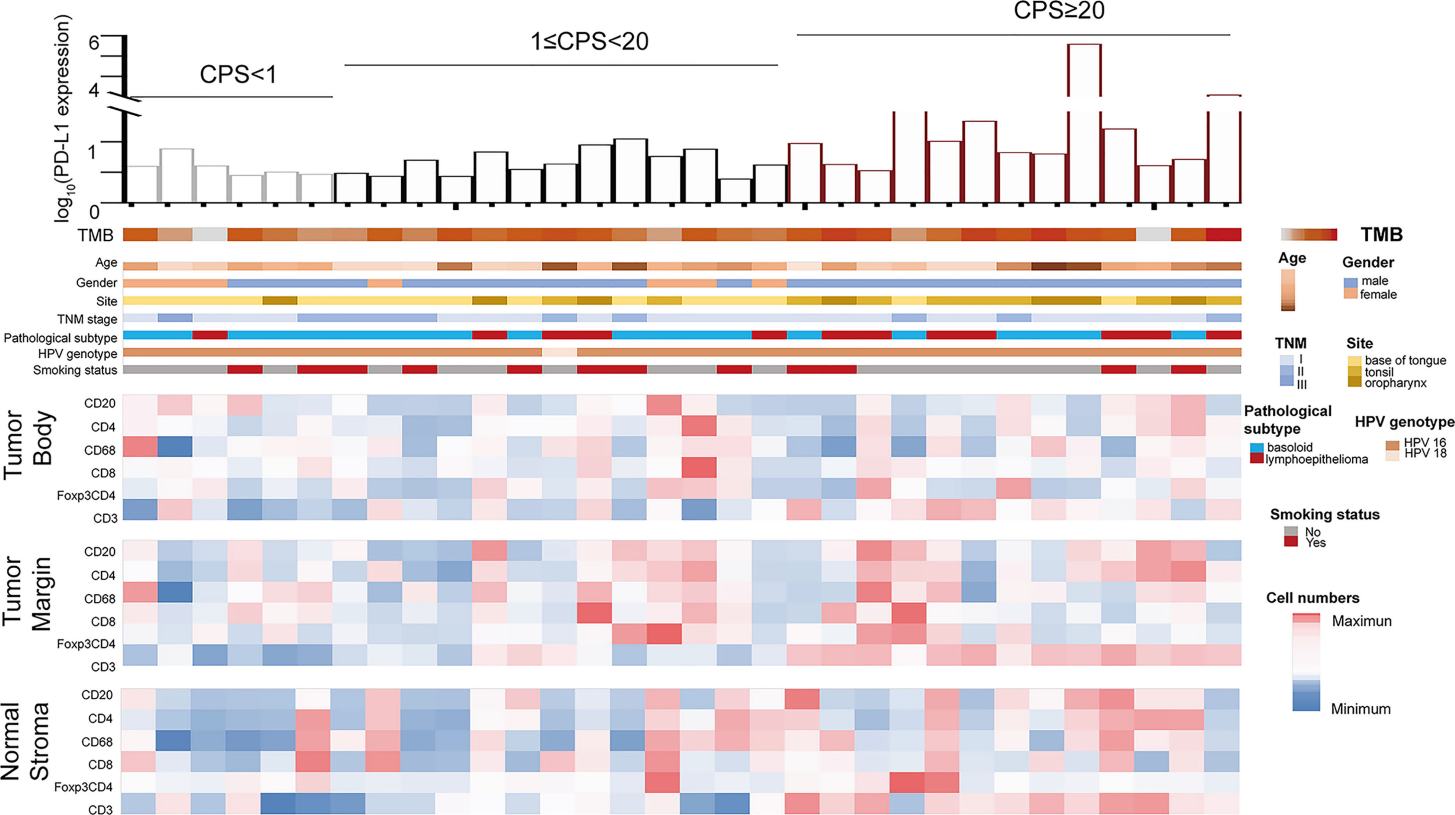
Overview of main findings from mIF, RNA sequencing, and WES analysis classified by CPS levels. Listed in descending order are PD-L1 RNA expression value, TMB, clinical parameters, mIF immune markers in TB, mIF immune markers in TM, mIF immune markers in NS.

**Figure 3:**
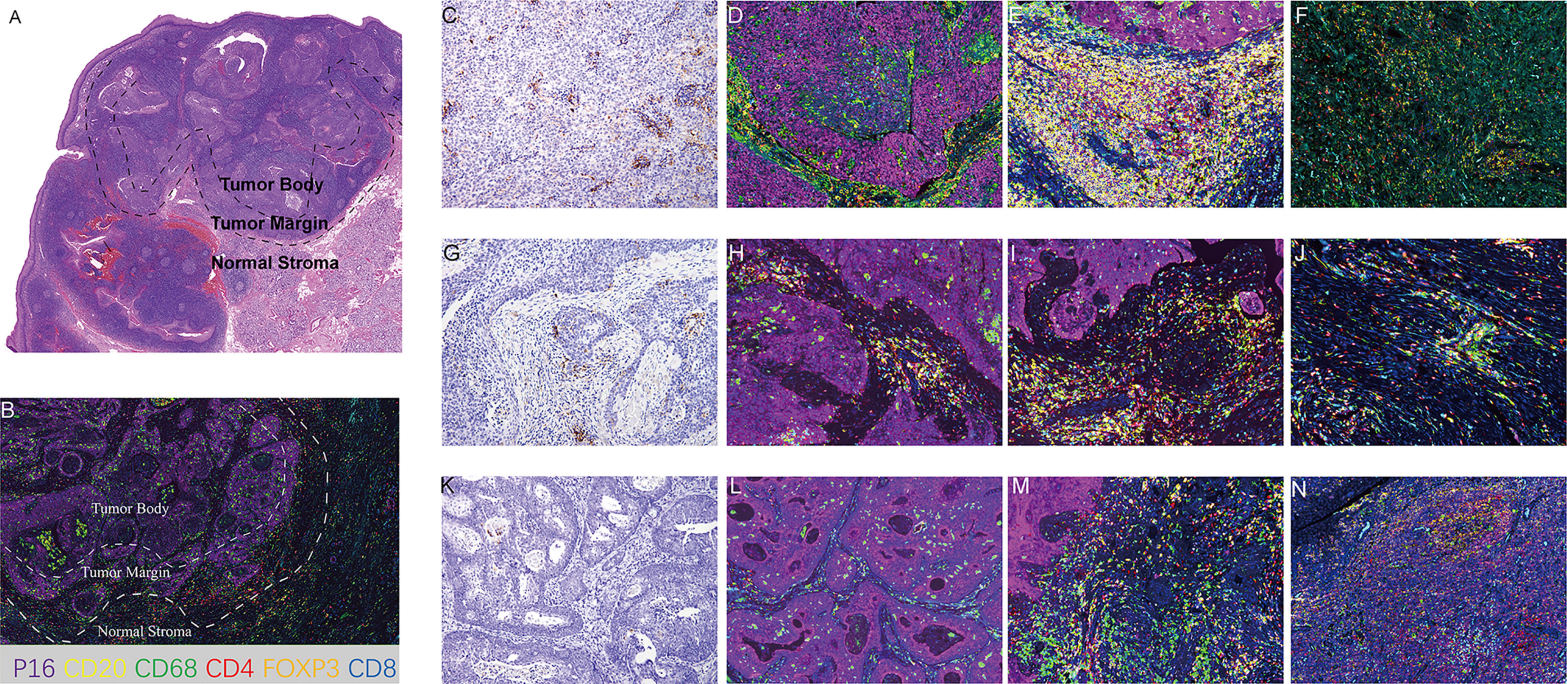
Representative images of mIF. **(A)** Definition of tumor body, tumor margin and normal stromal regions in HE section. **(B)** Definition of tumor body, tumor margin and normal stromal regions in mIF section. **(C–F)** Representative images of CPS≥20 cases. **(C)** PD-L1 staining (200X). **(D)** mIF in TB (200X). **(E)** mIF in TM (200X). **(F)** mIF in NS (200X). **(G–J)** Representative images of 1≤ CPS<20 cases. **(G)** PD-L1 staining (200X). **(H)** mIF in TB (200X). **(I)** mIF in TM (200X). **(J)** mIF in NS (200X). **(K–N)**. Representative images of CPS<1 cases. **(K)** PD-L1 staining (200X). **(L)** mIF in TB (200X). **(M)** mIF in TM (200X). **(N)** mIF in NS (200X). CD20, yellow; CD68 green; CD4 red; Foxp3, orange; CD8, blue; P16, purple; DAPI for nuclei.

**Figure 4:**
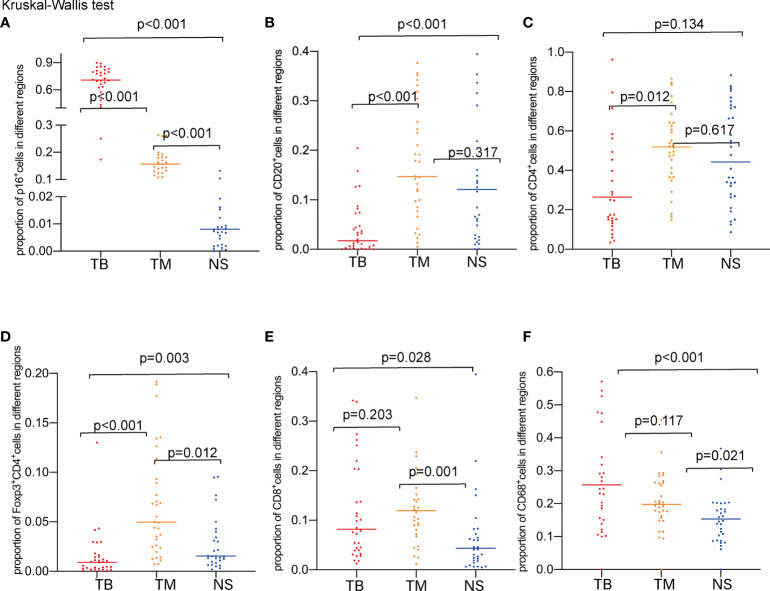
Proportions of marker-positive cells according to TB, TM, and NS regions. **(A)** Comparison of the proportions of P16^+^ cells. **(B)** Comparison of the proportions of CD20^+^ cells. **(C)** Comparison of the proportions of CD4^+^ cells. **(D)** Comparison of the proportions Foxp3^+^CD4^+^cells. **(E)** Comparison of the proportions of CD8^+^ cells. **(F)** Comparison of the proportions of CD68^+^ cells.

A total of 32 samples were selected for CD20, CD68, CD4, Foxp3, CD8, and P16 (for tumor cell staining) expression analysis by multiple immunofluorescence (mIF) staining and CD3 expression analysis by IHC staining. **Figure 2** depicts an overview of the 32 samples with mIF and their associated clinical and molecular profiles. Because intra-tumor heterogeneity was observed for the TILs and the immune response varied among the tumor regions, mIF data were analyzed individually for TB, TM, and NS regions as defined in **Figures 3A, B**, and TILs were classified as CD3^+^, CD20^+^, CD68^+^, CD4^+^, CD8^+^, and Foxp3^+^CD4^+^TILs. The typical images of mIF staining were illustrated in **Figures 3C–N**. Overall, the percentage of tumor cells that were stained by P16 differed significantly across the TB, TM, and NS regions (66.04% vs. 17.31% vs. 2.08% on average, p<0.001, **Figure 4A**), indicating that supportive regions were chosen appropriately. The proportion of each lymphocyte in each separate region was computed while excluding tumor cells, and we found that TM regions had significantly higher CD20^+^, CD4^+^ and Foxp3^+^CD4^+^ TILs infiltration than TB regions (p<0.001; p=0.012 and p<0.001, respectively, **Figures 4B–D**). The distribution of CD8^+^ and CD68^+^ TILs in the TB and TM regions were comparable, but less so in the NS regions (**Figures 4E, F**). In conclusion, CD20^+^, CD4^+^ and Foxp3^+^CD4^+^ TILs shared similar infiltration pattern, while CD8^+^ and CD68^+^ TILs infiltration patten were comparable.

**Figure 5:**
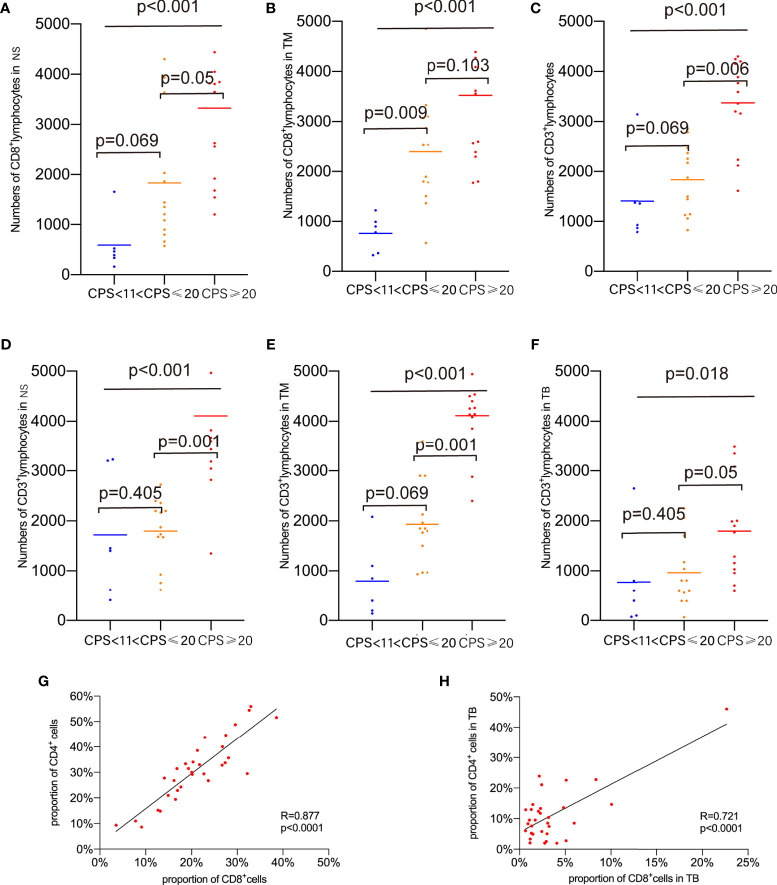
Proportions of marker-positive cells according to CPS levels. **(A)** Comparison of the proportion of CD8^+^cells in NS. **(B)** Comparison of the proportion of CD8^+^cells in TM. **(C)** Comparison of the proportion of CD3^+^cells in whole area of slides. **(D)** Comparison of the proportion of CD3^+^cells in NS. **(E)** Comparison of the proportion of CD3^+^cells in TM. **(F)** Comparison of the proportion of CD3^+^cells in TB. **(G)** Correlations of CD8^+^ cell and CD4^+^ cells in whole area of slides. **(H)** Correlations of CD8^+^ cell and CD4^+^ cells in TB.

To determine the association between TILs infiltration and CPS, the number of infiltrating lymphocytes was compared, and the results revealed that the number of CD8^+^TILs varied significantly in the NS and TM regions but not in the TB regions classified by methodology A (**Figures 5A, B**, both p<0.001). In the NS and TM regions, as previously analyzed, CD8^+^ lymphocyte infiltration correlated positively with CPS levels, with the PD-L1-H group showing slightly higher CD8^+^ lymphocyte infiltration than the PD-L1-M group in the TM region. Differences in classification were also found when using methodology B (p=0.001 in the NS region and p<0.001 in the TM region) and methodology C (p=0.001 in the NS region and p=0.004 in the TM region). In the PD-L1-H group, CD3^+^ TILs showed higher infiltration in the entire area of the slide, NS, TM and TB regions, indicating that TILs were more prevalent in patients with high PD-L1 expression (**Figures 5C–F**). Other TILs did not vary significantly across the various CPS groups in any of the TB, TM, or NS regions.

The correlations between immune marker proportions were also analyzed. The proportion of CD8^+^ TILs in the entire area of the slides (R=0.877, p<0.0001) and the TB region (R=0.721, P<0.0001) showed a strong positive correlation with CD4^+^ TILs as shown in **Figures 5G, H**. Foxp3^+^CD4^+^ TILs correlated with CD20^+^ TILs and CD4^+^ TILs in the NS, TB, and TM regions (with CD20^+^ TILs, R=0.548, p=0.0012 in NS; R=0.443, p=0.0112 in TB; R=0.429, p=0.0142 in TM, with CD4^+^ TILs, R=0.389, p=0.0278 in NS; R=0.599 p=0.0003 in TB; R=0.365, p=0.04 in TM, **Supplementary Figures S2A–F**). CD20^+^ TILs had positive relationships with CD4^+^ TILs infiltration in NS, TM, and entire slide regions (R=0.589, p=0.0004 in NS; R=0.744, p=0<0.0001 in TM; R=0.614, p=0.0002 in entire area of slides (**Supplementary Figures S2G–I**). There was no relationship found between CD68^+^ TILs and other TILs.

### Genomic Alterations and Mutation Signature Based on PD-L1 CPS

**Figure 6:**
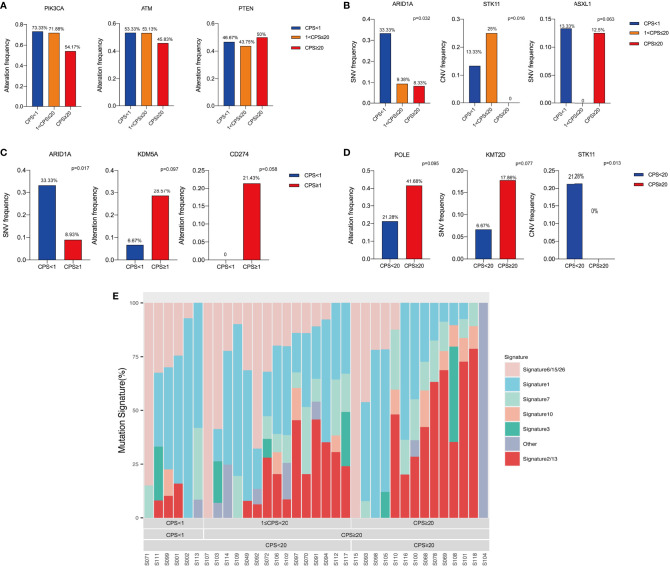
Genomic alterations analysis based on CPS levels. **(A)** The top common altered genes. **(B)** Altered genes among different CPS groups classified by methodology A. **(C)** Altered genes between different CPS groups classified by methodology B. **(D)** Altered genes between different CPS groups classified by methodology C. **(E)** Mutation signature analysis based on CPS levels.

Driven genomic alterations, including EGFR alterations and PD-L1 amplification, have been reported to be predictors of immunotherapy response (14). A panel of genes that have been shown to be associated with immunotherapies were selected to determine the genomic profiles of HPV(+)OPSCC in various CPS groups, and to characterize the immune-related genomic landscape of HPV(+)OPSCC based on CPS. According to the findings of the next-generation sequencing, HPV(+)OPSCC has distinct genetic characteristics in various CPS groups. The most common genetic changes in all groups were PIK3CA, ATM, and PTEN without significant differences (**Figure 6A**). However, when classified by methodology A, the rates of gene single-nucleotide variants (SNVs), such as ARID1A (p=0.032), and gene copy number variations (CNVs), such as STK11 (p=0.016), were highest in the PD-L1-L group compared with the PD-L1-M and PD-L1-H groups. At the same time, the SNV rate of ASXL1 varied among three CPS groups with marginal significance, with the PD-L1-M group not exhibiting SNV of ASXL1 (p=0.063) (**Figure 6B**). When we divided the whole group into CPS<1 and CPS≥1 using methodology B, we found that ARID1A had a lower SNV rate in CPS≥1 group(p=0.017), while KDM5A (p=0.097) and CD274 (p=0.058) demonstrated a higher genetic altering trend (including SNV and CNV) in the CPS≥1 group (**Figure 6C**). When the CPS cut-off value was set to 20, the PD-L1-H group showed lower STK11 (p=0.013) alterations, but higher POLE alterations (p=0.095) and KMT2D SNVs (p=0.077) with marginal significance (**Figure 6D**). In addition, a comparison was made of the distribution of all mutation signatures across the 32 cases that received WES and were classified based on PD-L1 expression (**Figure 6E**). The mutation signatures of APOBEC (apolipoprotein B mRNA editing enzyme, catalytic polypeptide-like, signatures 2 and 13) cytosine deaminase editing, which is associated with antiviral immunity, were significantly linked to CPS scores according to methodology A (Pearson’s correlation coefficient=0.417, p=0.013) and C positively (Pearson’s correlation coefficient=0.375, p=0.026, **Supplementary Figure S3A**), while no significant relationship was observed in other mutation signatures, including the dMMR/MSI signature (**Supplementary Figure S3B**).

### Variations in Immune Markers and Pathway Expression Based on PD-L1 CPS

**Figure 7:**
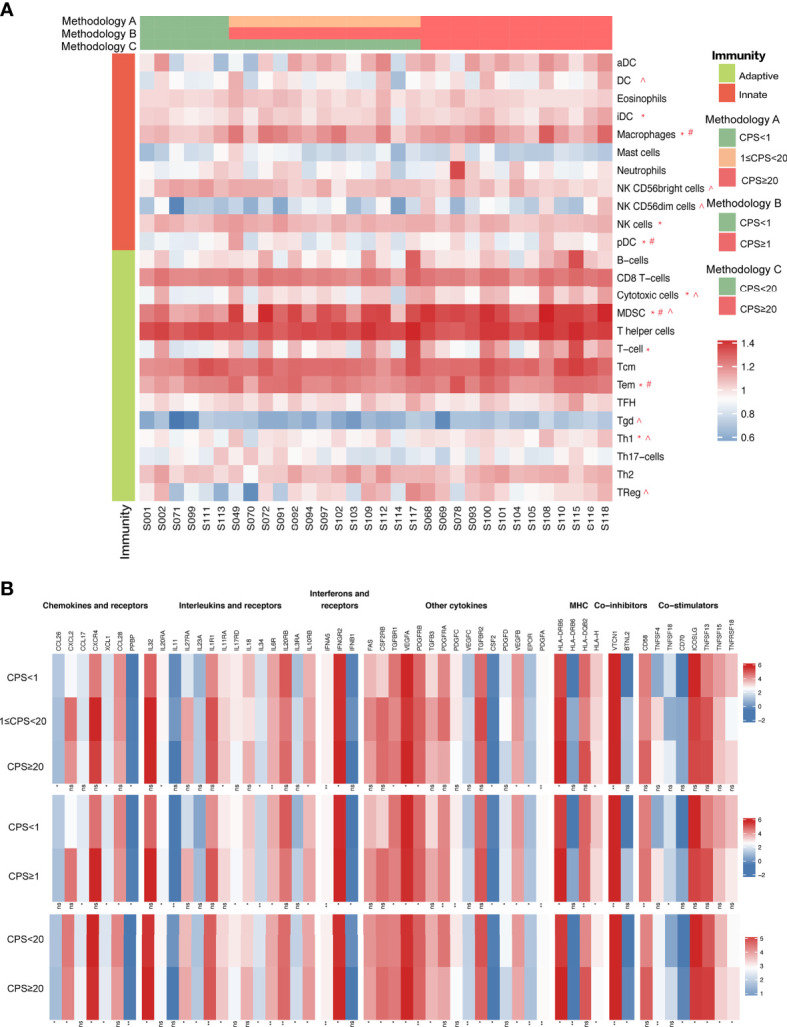
**(A)** Immune cell marker expression analysis on RNA-sequencing data based on CPS levels. **(B)** Immune signature analysis based on CPS levels. “*” indicates immune cell markers expressed significantly different classified by methodology A. “#” indicates immune cell markers expressed significantly different classified by methodology B. “^” indicates immune cell markers expressed significantly different classified by methodology C.

We examined the immunogenic characteristics based on PD-L1 CPS by performing differential immune cell marker expression analysis on RNA-sequencing data from 32 tumors. Overall, most immune markers were increased with PD-L1 expression except for natural killer (NK) CD56 bright cells, which exhibited a declining trend with CPS (**Figure 7A**). CPS levels enhanced the infiltration of myeloid-derived suppressor cells (MDSCs) independent of technique A, B, or C. We also saw an increase in immature dendritic cells (iDCs), macrophages, NK cells, plasmacytoid dendritic cells (pDCs), cytotoxic cells, T cells, Tem cells, and Th-1 cells with CPS levels defined by methodology A, whereas macrophages, pDCs, and Tem cells had higher expression defined by methodology B, and cytotoxic cells and Th-1 cell infiltration was increased together with the CPS when it was defined by methodology C. Notably, only when CPS=20 was used as the cut-off value did the expression of DCs, NK CD56dim cells, Tgd cells, and Treg cells exhibit a positive correlation with CPS. To be noticed, a positive correlation between PD-L1 RNA expression level and TMB was also observed (R^2^ = 0.1426, p=0.0331, **Supplementary Figure S4**). We performed immune-related gene pathway analysis on the 32 cases. Cases with different CPSs showed differential immune-related markers expression, including chemokines and receptors, interleukins and receptors, interferons and receptors, major histocompatibility complex (MHC), co-inhibitors, co-stimulators, and other cytokines as shown in **Figure 7B**. The detailed markers that positively and negatively correlated with CPS are illustrated in **Supplementary Table S1**.

## Discussion

PD-L1 expression has been identified as a marker of immune surveillance escape and has been linked to improved survival in HPV(+)OPSCC (15, 16). CPS, which assesses PD-L1 expression in both tumor and immune cells, has firmly established itself as a biomarker for predicting ICIs response, whereas distribution of CPS in HPV(+)OPSCC has not been comprehensively studied. Knowing the TIM, molecular characteristics and immune profile of HPV(+)OPSCC with various CPSs is critical for gaining understanding of immunotherapy effect mechanisms. In this study, we divided HPV(+)OPSCC patient samples into different PD-L1 expression levels scored by CPS and analyzed their clinicopathological characteristics, exploring their association with other immunotherapy biomarkers and tumor immune microenvironment. We also comprehensively examined their molecular features and immunomarkers expression in each PD-L1 group.

In comparison with HPV (-)HNSCC, of which 40% of the cases (140/359) were reported to have a CPS≥1, we had 73.2% (80/112) CPS≥1 cases, indicating higher PD-L1 expression in HPV(+)OPSCC than in HPV(-) counterparts (17). The unique tumor microenvironment of HPV(+)OPSCC, which is located in the tonsils mainly and surrounded by a high density of lymphocytes, may explain its higher PD-L1 expression. Additionally, our finding that tumors in the tonsils and TIL-rich subtypes had significantly higher CPS levels further supports this concept. Tobacco smoking has been identified as a major cause of HPV(-)HNSCC and has been linked to a decrease in the number of immune cells (18). Some studies have reported that smoking has an immunosuppressive effect, and current smokers have significantly lower numbers of PD-L1^+^ cells than non-smokers in HPV(-)HNSCC (18), which was also observed in our study, hence the effects of smoking on tumor immune microenvironment in HPV(+)OPSCC warrant further investigation. The prognostic role of PD-L1 in HNSCC varies according to different studies, with some studies reporting that it may predict improved survival, and others reporting the opposite (19), while high PD-L1 expression on >5% intra-tumoral immune cells together with high CD8^+^ TILs infiltration were shown to be significantly associated with better prognosis in HPV(+)OPSCC (10). Although the log-rank test did not reveal any significant differences in OS across CPS groups, we found that high PD-L1 levels showed a better prognosis trend, and its prognosis prediction role needs further investigation.

In 2020, the FDA approved pembrolizumab for the treatment of patients with advanced tumors and high TMB (≥10 mutations/Mb) who had no alternative treatment choices in melanoma, NSCLC, HNSCC, etc. (20). This approval validates TMB as a predictive biomarker of ICI efficacy, and the reason may be attributed to an increase in neoantigens, which allows for enhanced immunogenicity (20). However, a retrospective research found that TMB could not predict ICI response efficacy in head and neck cancers as CD8^+^ T cells were not positively associated with neoantigen load in this tumor (8). Because the study did not classify HNSCC based on HPV status, the immunotherapy efficacy predictive significance of TMB in HPV(+)OPSCC requires further investigation. In our study, CD8^+^ TILs correlated significantly with TMB, further supporting the immunotherapy response predictive role of TMB. TMB and CD8^+^ TILs were significantly greater in the PD-L1-H group, and this may be the reason why patients with increased PD-L1 expression respond better to ICIs. However, since our study was retrospective, a prospective study is warranted to further establish the relationship between PD-L1 and ICI efficacy in HPV(+)OPSCC.

Due to the existence of intra-tumoral heterogeneity, we divided the tumor into 3 distinct regions for comparison of different TILs using multiplex immunofluorescence staining. As anticipated, patients with higher PD-L1 expression had extensive lymphocyte infiltration, including CD3^+^ and CD8^+^ TILs. Different immune cells exhibit unique distributions throughout different regions, as CD20^+^, CD4^+^, and Foxp3^+^CD4^+^ TILs were enriched in TM regions, highlighting their regulatory role in immune function as reported (21), while CD8^+^ TILs were more enriched in the tumor regions (TB and TM) than in the NS, suggesting their cytotoxic roles in tumor cells. Notably, there was a strong positive correlation between CD8^+^ and CD4^+^ cells, particularly in the TB region, suggesting that CD4^+^ cells play a role when cytotoxic CD8^+^ cells are active. All in all, CD20^+^, CD4^+^ and Foxp3^+^CD4^+^ TILs, as well as CD8^+^ and CD68^+^ TILs had similar infiltration patten respectively, while the trend was different in HPV(-) HNSCC (18).

Mutation in the tumor suppressor gene STK11, which regulates cell proliferation, was shown to be a negative immunotherapy predictor in non-small cell lung carcinoma (NSCLC) patients, and STK11 mutations were associated with worse survival than those without mutations (22). STK11 alterations were shown to be less frequent in the PD-L1-H group in this study, confirming their negative predictive ability. Loss of ARID1A was reported to be associated with increasing TMB, TILs, and PD-L1 expression in ovarian clear cell carcinoma and gastric carcinoma (23–25), and patients with ARID1A deficiency had a higher response rate to immunotherapy (26). Conversely, we found a higher mutation rate of ARID1A in the PD-L1-L group; therefore, ARID1A alterations should be further confirmed as predictive biomarkers for immunotherapy in HPV(+)OPSCC. Similarly, POLE alterations were associated with increased TMB, CD8^+^ cytotoxic T cells and longer OS following immunotherapy, and patients with POLE mutations were selected for related clinical trials (27). Amplification of CD274, which encodes PD-L1, was similarly associated with immunotherapeutic sensitivity despite being less frequently detected in HNSCC (3.1% as reported) (14). POLE and CD274 alterations, both of which were favorable predictors of immunotherapy, were also enriched in the PD-L1-H group with marginal significance, and the lack of amplification of CD274 in the PD-L1-L group further confirmed the accuracy of this study. PTEN loss was one of the most frequent genomic alterations found in this study, and it conferred immunotherapy resistance in animal models (28), however, the levels of PTEN were not significantly different among the PD-L1 groups. Although KMT2D, KDM5A, and ASXL1 were not reported to be correlated with immunotherapy, the fact that they exhibited some distinct change based on CPS indicates that further research is needed to determine their impact on immunotherapeutic effects.

Genes of the APOBEC family, which may inhibit HPV infection activity, were found to be highly expressed after the anti-viral innate immune response was activated (29). At the same time, the APOBEC family may accelerate host genome mutagenesis, resulting in the accumulation of APOBEC mutation signatures (signatures 2 and 13). APOBEC family cytosine deaminase editing mutation signatures were found to be significantly higher in HPV(+)OPSCC than in HPV(-)HNSCC (30), suggesting that APOBEC editing and innate viral immune response are the principle drivers of HPV(+)OPSCC mutation. The APOBEC mutation signature was shown to be significantly more frequent in the PD-L1-H group herein. Notably, the POLE mutation, which is associated with APOBEC, also occurred more frequently in the PD-L1-H group than PD-L1-M and PD-L1-L group. As a result, the higher PD-L1 expression group was thought to have a greater immune evasion and innate immune response, increasing the APOBEC mutation signature and associated gene mutations. We also compared additional mutation signatures, including the dMMR/MSI mutation signature in various CPS groups and found no significant differences.

The analysis of immune signatures among different CPS groups classified by methodologies A, B, and C revealed several differentially expressed genes that have been linked to various functions in immune regulation. The chemokine XCL1 was shown to predict the response to pembrolizumab in NSCLC (31), while CCL17 could boost the Th2-type immune response (32), and both were positively associated with CPS. The genes in the PD-L1-H group that were expressed at lower levels exhibited a negative relation with immunotherapy. CCL26, for example, has been shown to be highly expressed in recurrent hepatocellular carcinoma (HCC) (33), while CXCL2 is highly expressed in STK11-mutated NSCLC, which has a poorer response rate to ICIs (34). Notably, STK11 mutations were rarer in the PD-L1-H group than the other two groups herein. Interleukins IL11 and IL11RA were shown to be more abundant in the higher CPS group classified by methodologies A and B respectively, and they were also found to serve as favorable predictors of immunotherapy and prognosis. IL11 was found to be positively associated with immune cell infiltration in HCC, while IL11RA was associated with a better prognosis in breast cancer (35). In terms of interferons and receptors, IFNGR2 conferred sensitivity to T-cell mediated cytotoxic activity and enhanced the anti-tumor effectiveness of CD8^+^ T cells *in vitro*, while high expression of IFNB1 was associated with chemotherapy resistance and a poor prognosis in glioblastoma (36, 37). Similarly, the cytokine TGFBR1, which is associated with PD-L1 expression in pancreatic adenocarcinoma, was shown to be highly expressed in the PD-L1-H group (1). Conversely, higher expression of cytokine CSF2, which was expressed at lower levels in the PD-L1-H group, predicts worse immunotherapeutic responses for stage III/IV melanoma patients (7). Therefore, the identification of differentially expressed genes in various CPS groups adds to our understanding of the immune microenvironment of HPV(+)OPSCC and offers prospective prognostic predictors or potential immunotherapy targets for future research.

Overall, 73.2% HPV(+)OPSCC had intermediate or high PD-L1 expression, and patients with higher PD-L1 expression tend to have better prognosis with marginal significance. Non-smoking patients, as well as those with tumors occurring at the tonsils or those that had rich TILs tended to show higher PD-L1 expression. Patients whose CPS≥20 had significantly higher TMB, and whose PD-L1 expression was positively associated with increased CD8^+^ TILs infiltration, supporting the idea that TMB-H could be used as an ICI efficacy biomarker in HPV(+)OPSCC. In mIF analysis, we found that PD-L1 expression reflected the immune-active status of the tumor, as the PD-L1-H group had higher T cell infiltration, especially in TB regions. The enrichment of ARID1A and STK11 alterations in low PD-L1 group may confer immune-suppression in this group. The enrichment trend of CD274 and POLE alterations in high PD-L1 group may be the reason for the immune-active status of the tumor. The APOBEC-associated mutation signature enriched in the PD-L1-H group also reflected higher innate viral immune responses in HPV(+)OPSCC. High expression of immune-related markers such as XCL1 and IL11 in the high PD-L1 group could explain the ICI efficacy prediction value of PD-L1. Overall, our study provides insight into the understanding of clinicopathological, tumor-immune-microenvironmental and molecular features of HPV(+)OPSCC based on PD-L1 expression and offers valuable information for deciding on a precise immunotherapeutic approach for HPV(+)OPSCC, as well as increasing understanding of immunotherapy mechanisms.

## Data Availability Statement

The datasets presented in this study can be found in online repositories. The names of the repository/repositories and accession number(s) can be found in the **Supplementary Material: Data sheet 1** **–** **12**.

## Ethics Statement

The study was approved by the Ethics Committee of Shanghai Ninth People’s Hospital (approval number: SH9H-2021-T493-1). Written informed consent for participation was not required for this study in accordance with the national legislation and the institutional requirements.

## Author Contributions

JL, and Z-YZ contributed to conception and design of the study. S-MX and C-JS organized the database. R-HX performed the statistical analysis. S-MX wrote the first draft of the manuscript. S-MX, C-JS, and R-HX wrote sections of the manuscript. All authors contributed to manuscript revision, read, and approved the submitted version.

## Funding

(1) Shanghai Municipal Key Clinical Specialty (shslczdzk01601). (2) Shanghai Clinical Research Center for Oral Diseases (19MC1910600). (3) Emerging Frontier Technology Joint Research Project (SHDC12018104). (4) Young Elite Scientist Sponsorship Program by the China Association for Science and Technology (grant 2019QNRC001).

## Conflict of Interest

The authors declare that the research was conducted in the absence of any commercial or financial relationships that could be construed as a potential conflict of interest.

## Publisher’s Note

All claims expressed in this article are solely those of the authors and do not necessarily represent those of their affiliated organizations, or those of the publisher, the editors and the reviewers. Any product that may be evaluated in this article, or claim that may be made by its manufacturer, is not guaranteed or endorsed by the publisher.

## Supplementary Material

Supplementary Table S1 Differently expressed immune-related markers based on CPS levels.

Supplementary Figure S1 Relationship of CD8+ TILs density and TMB.

Supplementary Figure S2 Association of different marker-positive cells in different regions. **(A)**. Association of CD20+cells and Foxp3+CD4+ cells in NS region. **(B)** Association of CD20+cells and Foxp3+CD4+ cells in TB region. **(C)** Association of CD20+cells and Foxp3+CD4+ cells in TM region. **(D)** Association of CD4+cells and Foxp3+CD4+ cells in NS region. **(E)** Association of CD4+cells and Foxp3+CD4+ cells in TB region. **(F)** Association of CD4+cells and Foxp3+CD4+ cells in TM region. **(G)** Association of CD20+cells and CD4+ cells in NS region. **(H)** Association of CD20+cells and CD4+ cells in TM region. **(I)** Association of CD20+cells and CD4+ cells in the entire area of the slide.

Supplementary Figure S3 The association of mutation signature and PD-L1 CPS levels. **(A)** Distinction of APOBEC mutation signature in different CPS groups. **(B)** Distinction of dMMR/MSI mutation signature in different CPS groups.

Supplementary Figure S4 Relationship between PD-L1 RNA expression value and TMB.

Supplementary Data Sheet 1 Gene list for genomic alteration analysis.

Supplementary Data Sheet 2 Chemokines and receptor expression and CPS.

Supplementary Data Sheet 3 Co-inhibitors expression and CPS.

Supplementary Data Sheet 4 Co-stimulators expression and CPS.

Supplementary Data Sheet 5 Interferon and receptor expression and CPS.

Supplementary Data Sheet 6 Interleukin expression and CPS.

Supplementary Data Sheet 7 MHC I expression and CPS.

Supplementary Data Sheet 8 MHC II expression and CPS.

Supplementary Data Sheet 9 Other cytokine expression and CPS.

Supplementary Data Sheet 10 Other MHC expression and CPS.

Supplementary Data Sheet 11 CPS and CD8+ TILs infiltration.

Supplementary Data Sheet 12 CPS and mutation signature analysis.
