# Supplementary material for: Analysis of Immunological Characteristics and Genomic Alterations in HPV-Positive Oropharyngeal Squamous Cell Carcinoma Based on PD-L1 Expression
Source: Front Immunol. 2022 Jan 25;12:798424. doi: 10.3389/fimmu.2021.798424 (PMC8821172; doi:10.3389/fimmu.2021.798424)
Supplement: Supplementary Table S1 — Differently expressed immune-related markers based on CPS levels. [file Table_1.docx]

**Supplementary Table S1. Differently expressed immune-related markers based on CPS levels**

|  | Genes whose expression levels correlate positively with CPS levels | | |  | Genes whose expression levels correlate negatively with CPS levels | | |
| --- | --- | --- | --- | --- | --- | --- | --- |
|  | classified by methodology A | classified by methodology B | classified by methodology C |  | classified by methodology A | classified by methodology B | classified by methodology C |
| Chemokines and receptors | XCL1 | XCL1 | XCL1 |  | CCL26 | CCL28 | CXCL2 |
|  | PPBP | PPBP | PPBP |  |  |  | CXCR4 |
|  |  | CCL17 |  |  |  |  |  |
|  | IL11RA | IL11 | IL27RA |  | IL34 | IL34 | IL34 |
| Interleukins and receptors | IL10RB | IL10RB | IL10RB |  | IL6R | IL6R | IL6R |
|  |  | IL18 | IL23A |  |  | IL17RD |  |
|  |  |  | IL1R1 |  |  |  |  |
|  |  |  | IL11RA |  |  |  |  |
|  |  |  | IL20RB |  |  |  |  |
|  |  |  | IL20RA |  |  |  |  |
|  | IFNA5 | IFNA5 | IFNA5 |  |  | IFNB1 |  |
| Interferon and receptors | IFNGR2 | INFGR2 | IFNGR2 |  |  |  |  |
|  | TGFBR1 | TGFBR1 | TGFBR1 |  | PDGFRB | PDGFRB | PDGFRB |
|  | VEGFA | VEGFA | VEGFA |  | CSF2 | CSF2 | CSF2 |
|  |  | PDGFRA | CSF2RB |  | VEGFB | VEGFB | VEGFB |
| Other cytokines |  | PDGFD |  |  | EPOR | EPOR | EPOR |
|  |  |  |  |  | PDGFA | PDGFA | PDGFA |
|  |  |  |  |  |  | PDGFC | FAS |
|  |  |  |  |  |  |  | TGFB3 |
|  |  |  |  |  |  |  | VEGFC |
|  |  |  |  |  |  |  | TGFBR2 |
|  | HLA-DRB5 | HLA-DRB5 | HLADRB5 |  | HLA-H | HLA-H | HLA-H |
| MHC |  | DQB2 | DRB6 |  |  |  |  |
| Co-inhibitor |  | VTCN1 | VTCN1 |  |  |  | BTNL2 |
|  |  | CD58 | TNFSF4 |  |  | TNFSF18 | ICOSLG |
|  |  |  | CD70 |  |  |  | TNFSF15 |
| Co-stimulator |  |  | TNFSF13 |  |  |  |  |
|  |  |  | TNFSF18 |  |  |  |  |
