# Supplementary figures and images for: Analysis of Immunological Characteristics and Genomic Alterations in HPV-Positive Oropharyngeal Squamous Cell Carcinoma Based on PD-L1 Expression
Source: Front Immunol. 2022 Jan 25;12:798424. doi: 10.3389/fimmu.2021.798424 (PMC8821172; doi:10.3389/fimmu.2021.798424)

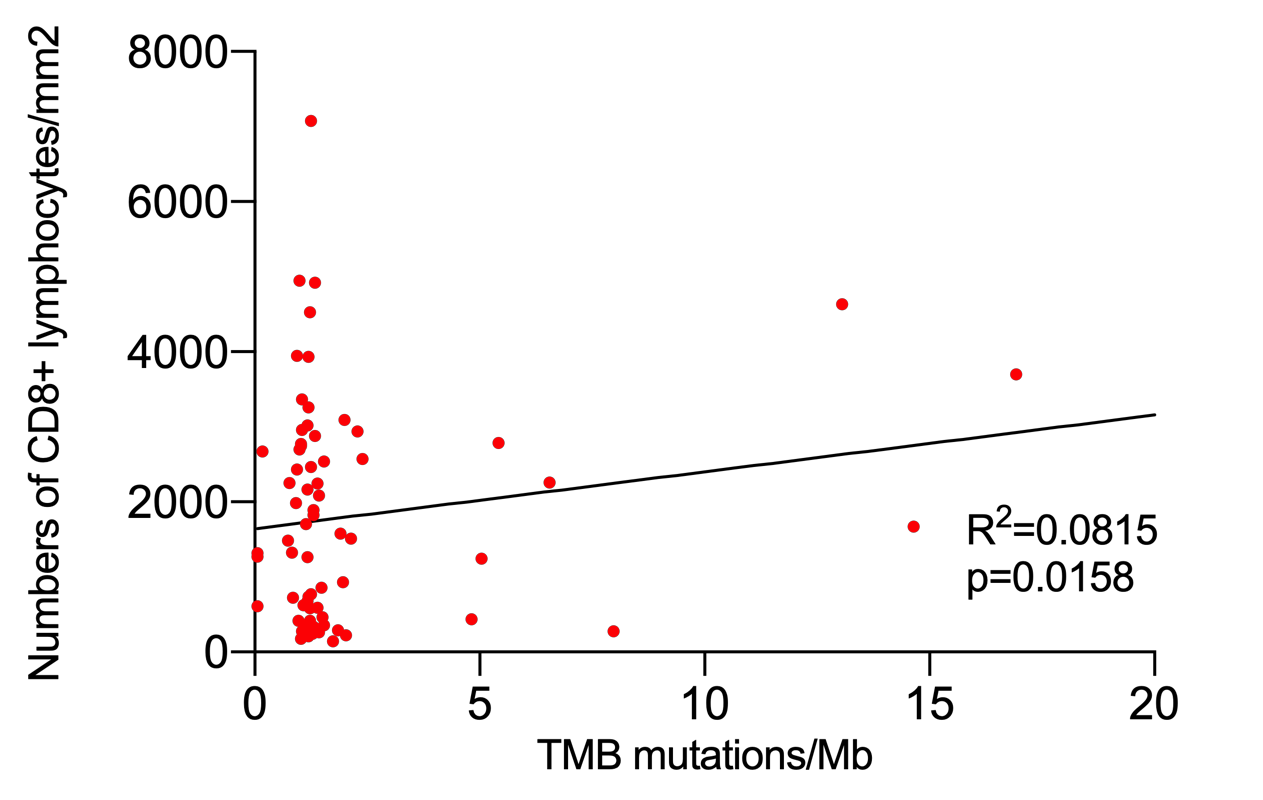

Supplement: Supplementary Figure S1 — Relationship of CD8+ TILs density and TMB. [file Image_1.tiff]

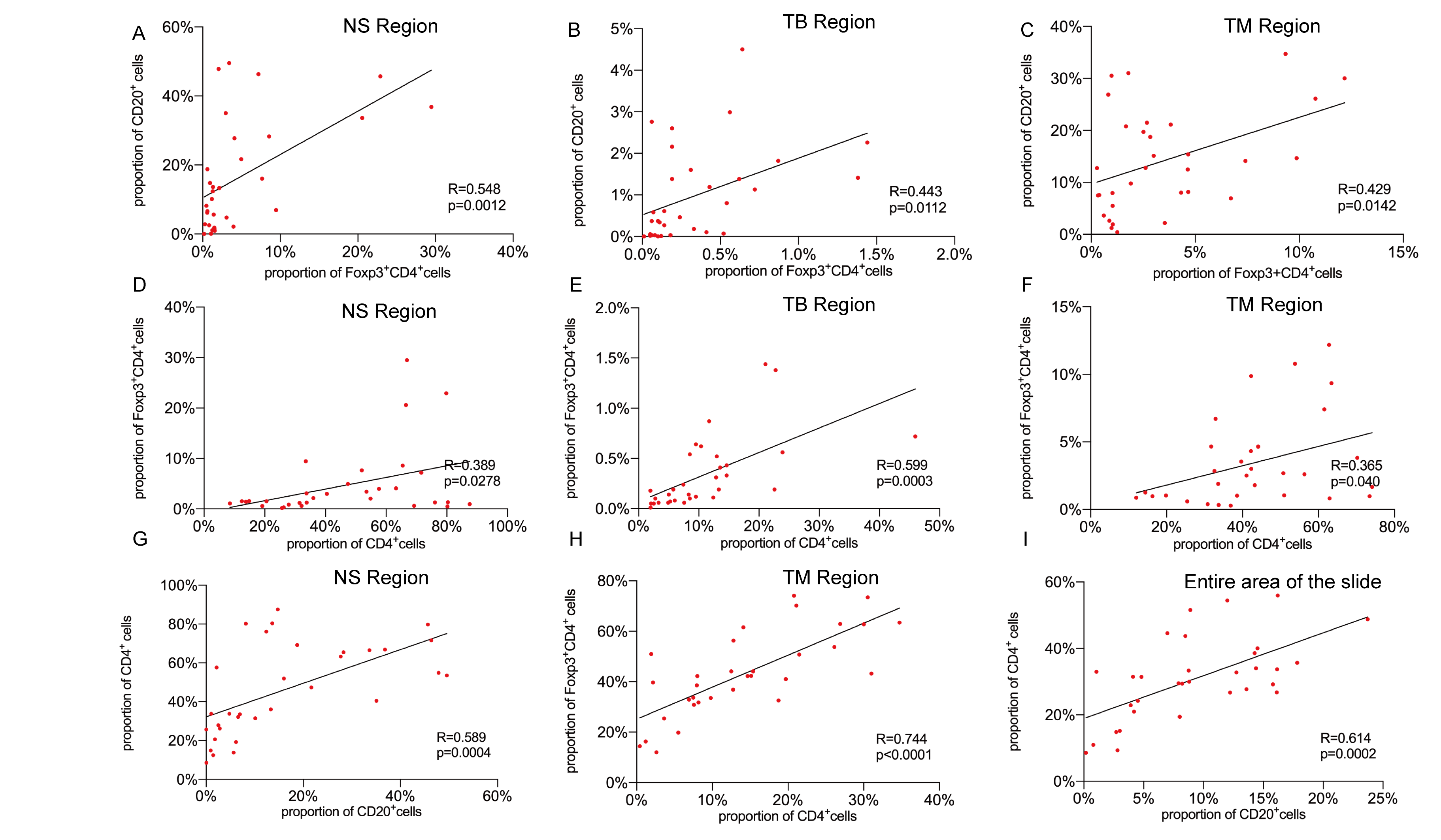

Supplement: Supplementary Figure S2 — Association of different marker-positive cells in different regions. (A). Association of CD20+cells and Foxp3+CD4+ cells in NS region. (B) Association of CD20+cells and Foxp3+CD4+ cells in TB region. (C) Association of CD20+cells and Foxp3+CD4+ cells in TM region. (D) Association of CD4+cells and Foxp3+CD4+ cells in NS region. (E) Association of CD4+cells and Foxp3+CD4+ cells in TB region. (F) Association of CD4+cells and Foxp3+CD4+ cells in TM region. (G) Association of CD20+cells and CD4+ cells in NS region. (H) Association of CD20+cells and CD4+ cells in TM region. (I) Association of CD20+cells and CD4+ cells in the entire area of the slide. [file Image_2.tif]

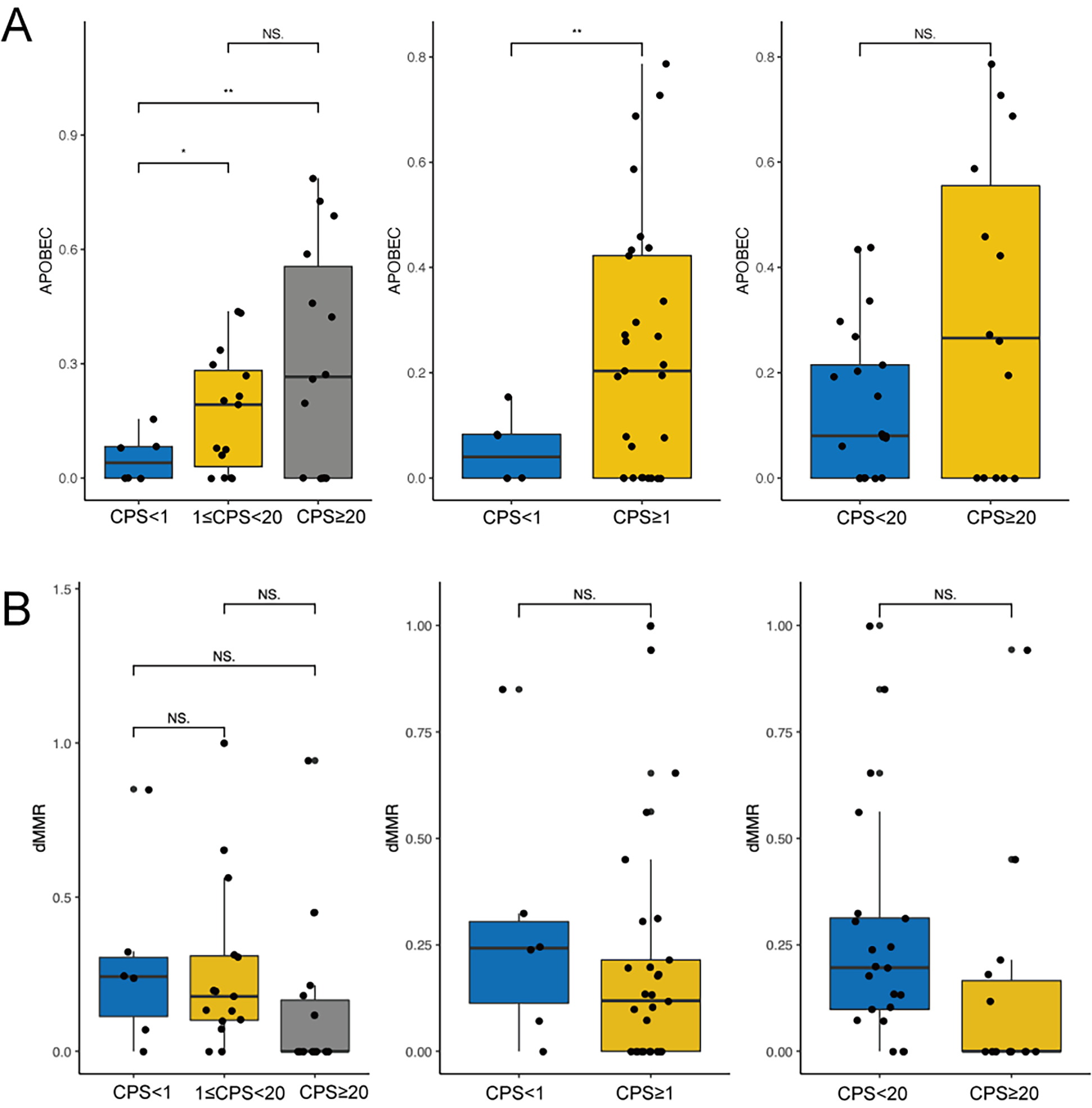

Supplement: Supplementary Figure S3 — The association of mutation signature and PD-L1 CPS levels. (A) Distinction of APOBEC mutation signature in different CPS groups. (B) Distinction of dMMR/MSI mutation signature in different CPS groups. [file Image_3.tif]

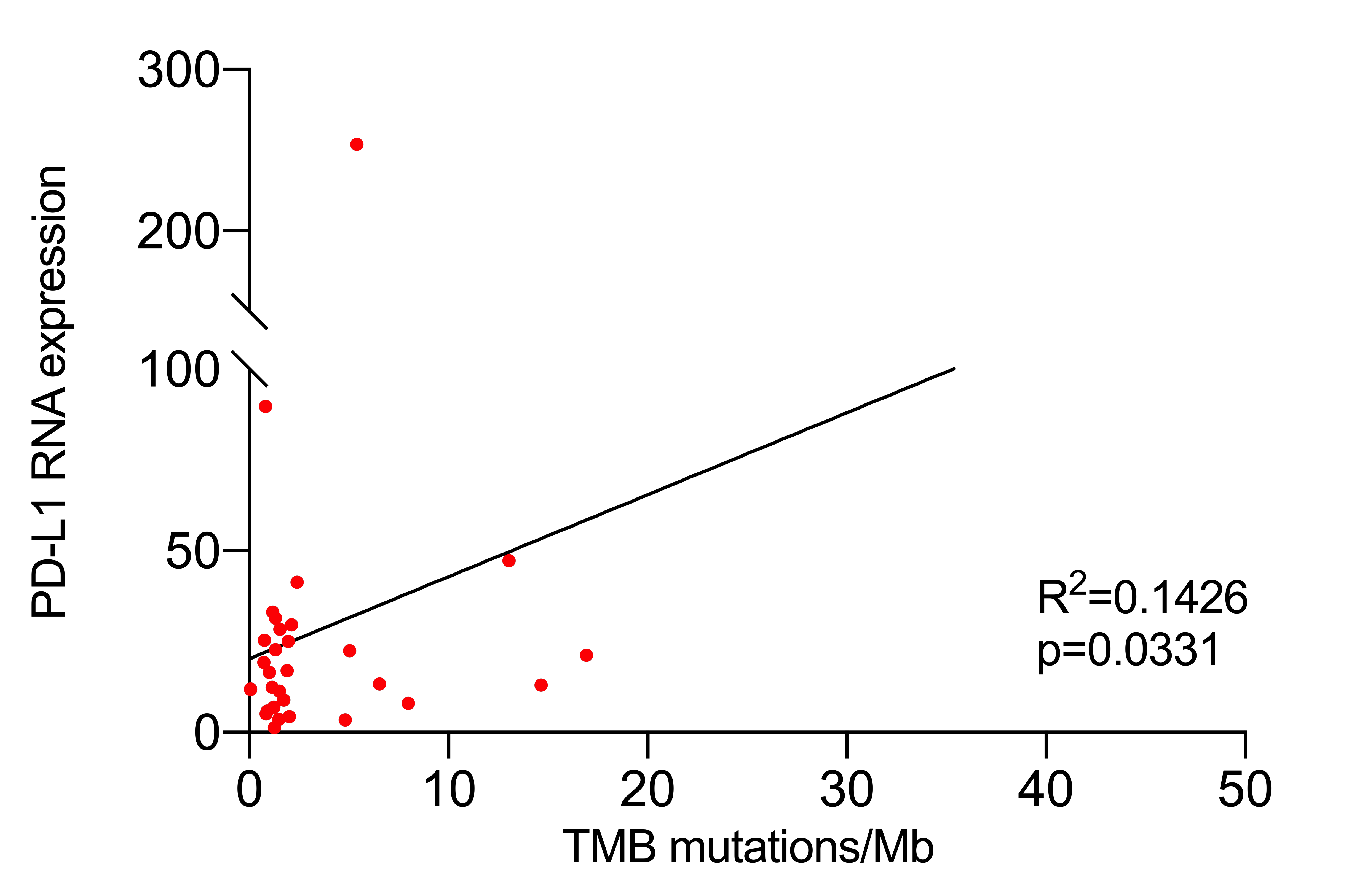

Supplement: Supplementary Figure S4 — Relationship between PD-L1 RNA expression value and TMB. [file Image_4.tif]
